# Supplementary material for: Acceptance and compliance with micronutrient powder (MNP) among children aged 6–23 months in northern Nigeria
Source: PLOS Glob Public Health. 2022 Oct 17;2(10):e0000961. doi: 10.1371/journal.pgph.0000961 (PMC10022258; doi:10.1371/journal.pgph.0000961)
Supplement: S1 Dataset — (ZIP) [file pgph.0000961.s008.zip › S8_Dataset/In-depth Interviews.zip!/In-depth Interviews/05072015_A_SO_AD_2_CG.pdf]

INTERVIEW WITH CAREGIVER AT MADUNGO COMMUNITY IN SONG LOCAL GOVERNMENT AREA, ADAMAWA STATE

I: Describe for me the people in your immediate family living in your household

R: There are four of us in this household. I, my husband and our two children.

I: Tell me more about your children

R: One is 3years 9months old and the other is 1year old

I: We have heard of food challenges for young children in this setting related to the season. Can you talk about these challenges please?

R: There were some people that came here to weigh our children but they didn’t talk about anything related to food. Is this what you mean?

I: I mean are there challenges related to food for young children in this community?

R: There are challenges related to food in this community; on getting adequate food. Sometimes there is shortage of food and other times after harvesting, before the food get to the house we experience some challenges.

I: Tell me more

R: Challenges related to the rainy season; the rain sometimes destroys our farms. So if we don’t harvest enough then there is always shortage of food before the next farming season.

I: Tell me more about how you cope with these shortages

R: It is not that bad because we always have what to give our children. Foods like groundnut and guinea corn are available throughout the year, it’s just foods like soya beans that are hard to come by here.

I: One thing we heard in other interviews is that caregivers often give their children 6-23months "tea". Could you define/explain "tea"?

R: sometimes I even give my 1year old child tea but he doesn’t like it much so I rarely give him.

I: Tell me more about "tea"

R: I give him Lipton with sugar. I don't add milk because I cannot afford it or any other beverage. If a child sees you drinking tea and wants to take some then there is no way you will refuse giving him. We are well enlightened or educated around here to know what should and should not be given to a small child; whatever we eat that is what we give our children. People in the clinic sometimes should guide us on such matters

I: Now I want us to talk about the MNP that you received. Could you tell me about your experience with the product?

R: We thank God that we got this product, but sometimes he doesn't like it and I have to feed him forcefully. If I forcefully give him sometimes he vomits the akamu

I: Tell me about the positive aspect of using MNP

R: Even though people said they have seen how my child has added weight, I don't see much change but when i carry him especially on my back, I feel the weight

I: Tell me more

R: He has increased appetite now because he eats better now unlike before that he will eat 4 or 5 spoonfuls only.

I: Uh-huh

R: When I started giving him the MNP, he experienced diarrhoea and I had to take him to the clinic, I have not even finished giving hi the drug yet.

I: You mentioned increased appetite as one of the benefits of using MNP, Can you tell me more please

R: He is more energetic and plays more with other children. I have really seen improvement on how he eats and there is a sign of vigour in his activities. Before I started using this MNP, he rarely eats and he easily gets tired when playing. As for the diarrhoea, it was after almost a week of starting the MNP that he started purging that is why I didn't pay much attention to it being associated with the MNP. We were given anti-diarrhoea but after 2days it didn't stop. He was purging about 2 to 3times in a day even though it was not as watery as the first few days. So I went back to the clinic where they prescribed anti malaria, diastop and ORS. He is better now but vomits when I forcefully feed him

I: Could you tell me any specific barrier that made it hard to use the MNP appropriately

R: There is no barrier in using the MNP. It's just the diarrhoea he had but I don't think that is a barrier because I didn't stop using it when it started. Another thing is that I have to forcefully give him the akamu with MNP most of the time

I: Some caregivers mentioned that it was hard to store the MNP without it being taken by others or eaten by rodents. Did you have any similar experience?

R: I don't a problem of storing because I keep it in my small suitcase (kit)

I: Some mothers indicated that it might be hard to remember to use MNP. Did you have a similar experience and what did you do to remember?

R: There is a problem because some of us mothers are careless or just lazy when it comes to taking care of the child. She won't heed to giving a child what will benefit him/her

I: Did you have a similar experience in forgetting to administer the MNP?

R: Well there are times ii forget to give him in the which is when I usually give him with Kunu, but I remember before the end of the day, usually forenoon or afternoon but I always give him everyday

I: Please tell me how you have been able to remember in cases where you forget in the morning

R: I remember based on the fact that I am used to giving him the MNP now

I: Could you tell me how we can address the challenge of remembering to use the MNP to ensure compliance and appropriate use?

R: Caregivers need to pay attention to their children very well.

I: Tell me more

R: As for you people that bring this MNP to us, we will really appreciate it if you will enlighten caregivers to care more about their children because sometimes forgetting this kind of things is due to lack of paying attention to the child.

I: Let's talk about the child's feeding and response to the MNP. Could you tell me the food with which you mix the MNP?

R: I mix it with kunun akamu.

I: Tell me more about the akamu

R: I don’t make it with groundnut. I just make plain akamu because we were told to mix it in kunu. Since kunun akamu is very soft and that is what I gives him most of the time

I: Could you tell me to what extent the child likes or dislike the taste of MNP?

R: Since I started giving him this MNP, it’s only once he drank it all without me forcing him. Even that one time, he didn’t finish the whole kunu so i had to reduce the quantity of kunu I mix the MNP in. That way he doesn’t vomit.

I: Tell me more

R: It seems he doesn’t like the taste of the MNP because when I add it in the kunu, it always smells like palm oil and taste like it.

I: Uh-huh

R: I had tasted it the first time I mixed it. It smells like palm oil and the taste is not bad because if I were to classify it I will say it doesn’t have any taste

I: Could you tell me how consumption of MNP has impacted the child's diet

R: Sincerely, my child's appetite has really increased since I started giving him MNP. I really appreciate this product. He consumes more breast milk too and some of the foods he didn’t like eating before, now he eats more varieties of food

I: In other communities we noticed that sharing of MNP occurred. Could you talk about any sharing of the MNP when you used it?

R: He has only one older brother and he has never asked to be given the MNP. He thinks it is a medicine and he doesn’t like medicine at all. The moment he sees me with the MNP he just leaves to play with his peers in front of the house and the other children in the community don’t come in when i administer it

I: Could you tell me if the leftover of the MNP is consumed by others?

R: i always ensure he finishes the kunun akamu that is mixed with MNP. There has never been leftover to be eaten by others.

I: Could you tell me whether 60sachet will be enough for 60days? Why?

R: We were told one sachet daily. Is that not right?

I: Yes it is one sachet per day. Could you tell me whether 60 sachets will be enough for 60days?

R: I have seen its importance and benefit to my child so if I am given me i will willingly collect it

I: Uh-huh

R: R: I heard of a caregiver in Bolki who got some empty sachet in the packet, in such cases there should be provision for extra ones to complete the 60days. Since those who made this MNP said it is meant for 60days, so i believe they know the importance of doing that. My suggestion is that there should be a provision for such cases so that it will be administered for the 60days as prescribed. As for me all the sachets i used were intact

I: Now that you have used the MNP, could you talk about how you perceive it?

R: It is good for the child because it brings so much improvement in the child's growth

I: could you tell me whether you see it as a food, medicine or something else?

R: When it was brought to us, we were told it is a body building food and full of nutrients.

I: Ok. Now that you have used it, how do you perceive it?

R: It is both food and medicine

I: uh-huh

R: my reason for saying this is my child is healthier and has more energy. He has increased appetite

I: Uh-huh

R: These are the major reasons why i said it is both food and medicine

I: Could you tell me whether you would purchase this product if it were cheaply on sale in this community?

R: Well if it is sold and I have the money i will be willing to purchase this product. This is because I have seen the importance and benefits and I won't feel bad buying it if I have the money.

I: Could you tell me how much you will be willing to pay for each sachet?

R: I can’t say it is those making it that can say how much should be paid. The way it is given for free now is okay, but if it were to be sold then 200Naira is a fair price for each packet.

I: You are giving me great information. Now please tell me some ways that you believe would be effective for promoting MNP to caregivers in this community so that they will comply with using it appropriately?

R: If you bring the MNP to the rest of the community just as your people came and gave us the MNP, the caregivers will be called and they will attend the gathering. Female caregivers need adequate enlightenment because some people are negligent when it comes to these kinds of things. I am surprised with myself too, I didn’t think I could do it daily because it is not that my child is ill and I have been administering it diligently. Enlightenment is really the answer.

I: I: Tell me more

R: I think this is the only way that caregivers will fully understand this product. It is during this enlightenment that the emphasis will be placed on how they should pay attention to what will benefit their child

I: Could you tell me who influences your decision regarding feeding your baby MNP?

R: My husband has been supportive. He encourages me to give the MNP and sometimes remind me before he goes out to farm. The day the MNP was given to us, he was in the farm. When he got back I showed him and told him about it; his father was present. I asked his opinion on it and he said since iit is for our child's improvement, try it. They told us you will come for other work and if we have any challenge or issues we should inform you.

I: Could you tell me a good place where health information related to MNP can be disseminated?

R: The best is to call a gathering of caregivers especially during distribution; they could be given the necessary information

I: Tell me more

R: Information can also be shared in mosque, church or the community leader’s premise. The day this MNP was brought to us, it was distributed in front our houses. There is a health worker here that is my father-in-law. He

works in the nearby clinic. If people are gathered here in front of his house they will listen to him because the community members trust and respect him

I: Tell me more

R: His name is Sani and he works in the nearby clinic. We trust him when it comes to receiving message especially about infant and children

I: Could you tell me what form of media you prefer for receiving MNP message?

R: When the MNP was distributed, we were given flyers. The best way to pass information about this MNP is during distribution when the caregivers are gathered in a specific location. Another way is through the word-of-mouth because most of the community members are not literate and cannot read what's on the flyer and might not think to ask someone who is to read it for them. Enlightenment through radio too is good because so many people even beyond this community will know about it. We also have town announcers who can go around ensuring people are gathered

I: Please describe for me a specific example of other successful health education campaigns in this community

R: There is immunization. When it comes to immunization, women always come out for it.

I: Uh-huh

R: In this community, anytime immunization will take place, the community leader tells the town announcer to go around and inform people of the community a day before the exercise. We gather at the school with our children to receive the immunization. Even though most of the women in this community are farmers, they always stay back whenever they hear about the immunization. It’s only a few people that I know that don’t allow immunization in their houses, one example is our neighbour he doesn’t let his children to be immunized

I: Could you tell me the best community channel to distribute the product?

R: in my opinion, the best way is to give the health worker around here because most of the people here are farmers and will not be met at home. Then he will inform the town announcer to tell people to come and collect it. The day this MNP was brought to us they met only a few of us because the people were not informed beforehand about it

I: I want to hear specific messages you think would effectively promote the product

R: I will inform people on the benefits i saw from experience. If the women are being reluctant to accept it then I will tell them "this product will increase your child's strength and energy and it has body building minerals in it. It will also boost your child's immunity and improve the child's appetite (my child breastfeeds more now and he is very active). This is an important product in a child's life and caregivers should accept it wholly"

I: Please explain to me how this product can be promoted but ensure sharing is limited

R: The people that brought this product know who is most appropriate to give the MNP that is why we should heed to their advice against sharing. The problem is even if it is shared, it will not benefit either of the parties given, the one that is supposed to be given and the others.

I: Finally, is there anything else that you can share about your experience with this product?

R: Well what i will like to say is about some of the people that get empty sachet, if it is mandatory for a child to take the MNP for 60days, then there should be provision for extra to make it complete. But if it is not necessary to take the MNP for 60days, then no problem.

I: Uh-huh

R: Women should be well enlightened about this MNP to ensure they accept it and use it appropriately. This is something that will benefit our children and a healthy child is the goal of every mother. Furthermore, if a mother gives a child this MNP and sees its benefits, she will be eager to get more even if it put in the market. The problem now is some people have not gotten this product so they don’t know the importance yet and might not buy. For example this RUTF (Ready to Use Therapeutic Food) i have heard that some buy it because they know the use and importance. Distribution should be through healthcare workers or it could be given to the community leaders and he will make the town announcer inform everyone. This is better than just coming here and not meeting anyone around because they have gone to farm or the forest and some women need their husband's permission to go out.

I: Can you recommend anything else to develop and effective program?

R: If there is enough enlightenment, then it is up to the women to put in their effort and ensure they use it appropriately. I can testify to the benefits of this MNP as i have seen improvement in my child. I think all women deserve to reap the benefit of this MNP but it is only when it is provided that they can use it, please ensure distribute it everywhere so that they will enjoy it too.

I:
